# GABAergic Input Affects Intracellular Calcium Levels in Developing Granule Cells of Adult Rat Hippocampus

**DOI:** 10.3390/ijms21051715

**Published:** 2020-03-03

**Authors:** Davide Lattanzi, Michael Di Palma, Riccardo Cuppini, Patrizia Ambrogini

**Affiliations:** Department of Biomolecular Sciences, University of Urbino Carlo Bo, I-61029 Urbino, Italy; davide.lattanzi@uniurb.it (D.L.); michael.dipalma@uniurb.it (M.D.P.); riccardo.cuppini@uniurb.it (R.C.)

**Keywords:** adult rat, GABA input, T-type voltage-dependent calcium channels, calcium-activated potassium channels, hippocampus, immature neurons, membrane potential oscillations

## Abstract

In the dentate gyrus (DG) of the mammalian hippocampus, granule neurons are generated from neural stem cells (NSCs) throughout the life span and are integrated into the hippocampal network. Adult DG neurogenesis is regulated by multiple intrinsic and extrinsic factors that control NSC proliferation, maintenance, and differentiation into mature neurons. γ-Aminobutyric acid (GABA), released by local interneurons, regulates the development of neurons born in adulthood by activating extrasynaptic and synaptic GABA_A_ receptors. In the present work, patch-clamp and calcium imaging techniques were used to record very immature granule cells of adult rat dentate gyrus for investigating the actual role of GABA_A_ receptor activation in intracellular calcium level regulation at an early stage of maturation. Our findings highlight a novel molecular and electrophysiological mechanism, involving calcium-activated potassium channels (BK) and T-type voltage-dependent calcium channels, through which GABA fine-tunes intracellular calcium homeostasis in rat adult-born granule neurons early during their maturation. This mechanism might be instrumental in promoting newborn cell survival.

## 1. Introduction

The hippocampus dentate gyrus (DG) subgranular zone (SGZ) represents one of the neurogenic niches where radial glia-like neural stem cells (NSCs) continuously generate new neurons throughout adulthood. This process, known as adult neurogenesis, can be outlined as the stepwise progression of NSCs into progenitor cells, neuroblast fate specification, neuronal differentiation in dentate granule cells (GCs), survival, and their synaptic integration into the existing circuitry to participate in the hippocampal function [1,2].

Neural activity affects multiple stages of hippocampal adult neurogenesis, and it is well established that the γ-aminobutyric acid (GABA) neurotransmitter plays a major role in mediating activity-dependent regulation of the neuronal development process, considering the early expression of GABA receptors (GABARs) by NSCs [3]. Indeed, GABA, probably released by dentate parvalbumin-expressing interneurons, regulates NSC quiescence [4], neuronal fate specification [5], and synaptic neuron integration in the hippocampal circuit [6]. Its functions are first mediated by tonic activation of extrasynaptic ionotropic GABA_A_ receptors onto newborn cells, and by phasic GABA activity when differentiating neurons receive GABAergic synaptic inputs from inhibitory interneurons of the subgranular zone and hilus [7].

Tonic and phasic GABA activity on maturating neurons generate membrane depolarizations due to an increased intracellular chloride concentration produced by high and low expression of Na^+^-K^+^-Cl^-^ co-transporters (NKCC1) and K^+^-Cl^-^ co-transporters (KCC2), respectively; as the neuronal maturation proceeds, a downregulation of NKCC1 and upregulation of KCC2 cotransporters occurs, thus dropping internal chloride concentration and making GABAergic signals hyperpolarizing [8]. The depolarizing activity of GABA onto adult-generated granule cells seems to be crucial for their maturation, probably due to calcium entry through low-voltage activated T-type Ca^2+^ channels (T-type VDCC) [6] precociously expressed by newborn cells.

In this context, we previously identified, in adult rat dentate gyrus, newborn granule cells at different stages of maturation, which exhibited peculiar morphological and electrophysiological properties [2] and synaptic input as well [9]. In line with literature evidence [10], our findings showed that at a very early stage of newborn neuron maturation, the stimulation of medial perforant pathway (MPP) or hilus did not evoke any detectable synaptic response, indicating the absence of functional synapses. At eight-day post-mitosis, developing neurons exhibited GABAergic synaptic input, eliciting small amplitude responses with slow kinetics, and T-type VDCC expression in the cell membrane (maturation stage reported as Class 2 subclass I in [2]). However, these very immature neurons also showed depolarized membrane potentials, likely inducing T-type VDCC inactivation, thus suggesting that GABA activity could not be able to promote increased calcium entry through T-type VDCC, as instead described for more mature newborn granule cells [10].

Additionally, we observed, during current-clamp recordings of newborn neurons at this very early maturation stage, the occurrence of resting membrane potential oscillations were not induced by synaptic activities, but likely spontaneously originated (unpublished data). Compelling evidence in literature points out that voltage-dependent calcium channels and calcium-activated potassium channels cooperate to generate robust membrane potential oscillations in several experimental models, thus regulating the intracellular calcium concentration [11,12,13].

Taking into account the above findings, we hypothesize that the spontaneous membrane potential oscillations recorded in 8-day old newborn granule cells (identified as Class 2 subclass I in [2]) may promote calcium entry and that GABA_A_R opening contributes to intracellular calcium level regulation, decreasing input resistance and dampening membrane potential oscillations. To address this issue, in the present paper, patch-clamp and calcium imaging techniques are used to record very immature granule cells of adult rat dentate gyrus. Our findings highlight a novel molecular and electrophysiological mechanism through which GABA fine-tunes intracellular calcium homeostasis in rat adult-born granule neurons at a very early stage of maturation. 

## 2. Results

### 2.1. Functional Features of Immature Dentate Granule Cells with GABAergic Evoked Response

**Figure 1:**
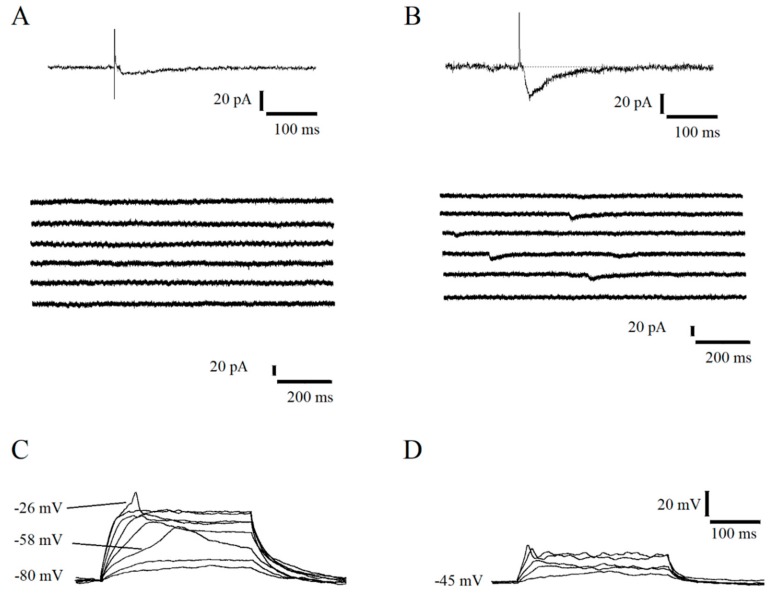
Functional features of immature dentate granule cells recorded in whole-cell configuration. (**A**,**B**) Voltage-clamp recordings (V-Holding = −70 mV, [Cl^−^]_i_ = 34 mM) of the evoked response to MPP stimulation (top) and spontaneous activity (bottom) in two different immature neurons. (**C**,**D**) Current-clamp recordings of intrinsic excitability in response to depolarizing current steps in the same recorded cell. TTX-sensitive rudimentary spike (threshold about −26 mV), and isolated and very slow Ni^2+^-sensitive spikes (threshold about −58 mV) were elicited by depolarizing current steps starting from membrane potential of −80 mV (**C**). Depolarizing current steps starting from resting membrane potential (−45 mV) were not able to elicit T-type VDCC spike due to their inactivation (**D**).

Immature granule cells showing only the GABAergic evoked response were considered (previously classified as Class 2 subclass I, [2]: RMP = -51.8 ± 0.9 mV; IR = 5888.6 ± 299.1 MΩ; C = 20.1 ± 0.7 pF). Most of the recorded cells exhibited a GABAergic response to MPP stimulation with low amplitude and slow kinetic, and no spontaneous events (Figure 1A); only a few cells elicited a GABAergic response with faster kinetic and higher amplitude and a very low-frequency GABAergic spontaneous synaptic activity as well (Figure 1B). According to our previous works [2,9,14], MPP-evoked responses and spontaneous activities were fully and reversibly abolished by 10 µM BMI, indicating the implication of ionotropic GABA_A_ receptors. The lack of any evoked glutamatergic currents was confirmed by recordings cells at V-_holding_ = −70 mV and +40 mV at maximal stimulation. Gramicidin perforated patch-clamp (*n* = 4 cells) proved the depolarizing nature of GABA_A_R currents elicited by 10 µM muscimol local application (reversal potential measured = -35.6 ± 4.2).

Depolarizing current injection in current-clamp mode, starting from a resting membrane potential of −80 mV, induced three different kinds of voltage-sensitive non-linearities: (i) a potassium outward current; (ii) a high threshold TTX (500 nM) sensitive rudimentary spike; (iii) a low threshold verapamil (20 µM) or Ni^2+^ (100 µM) sensitive slow spike. The low threshold activation and the sensibility to verapamil and nickel indicated that slow spikes were mediated by low-threshold T-type VDCC (Figure 1C). As expected, when the cells were depolarized starting from their resting membrane potential, it was not possible to elicit calcium spikes due to T-type VDCC inactivation (Figure 1D). In line with this, calcium spikes showed an activation threshold (−64.5 ± 2.1 mV) more hyperpolarized with respect to cell resting membrane potential (−52.8 ± 1.7 mV; *n* = 8 cells, paired T-test *p* < 0.001).

**Figure 2:**
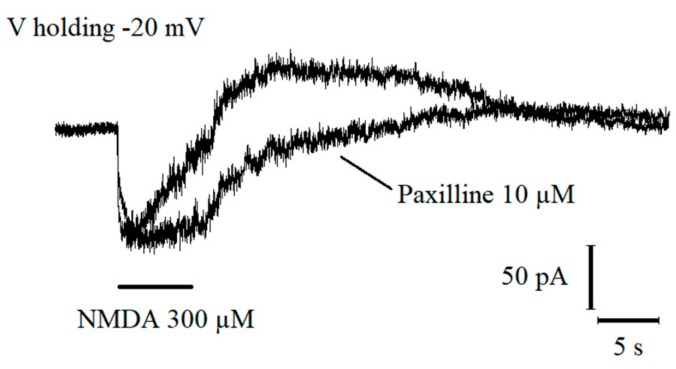
NMDA-induced BK channel activation via NMDARs in immature granule cells. Representative NMDA-induced current at −20 mV holding potential before and after paxilline 10 µM bath application.

Glutamate receptor expression was investigated by glutamate (100 µM) or N-Methyl-D-Aspartate (NMDA; 300 µM) application through micro-puffer apparatus and, consistent with our previous findings [15], an inward current was elicited when the cell was clamped at −80 mV. At more depolarized holding potentials (−20 mV), the inward current was followed by a robust outward current persisting after the end of glutamate application, which was blocked by TEA (2 mM) or Paxilline (10 µM) (Figure 2). The high paxilline sensibility and the voltage dependence indicated that the outward current was mediated by BK-type Ca^2+^-activated potassium channels.

### 2.2. Resting Membrane Potential Oscillations and Intracellular Ca^2+^ Levels

**Figure 3:**
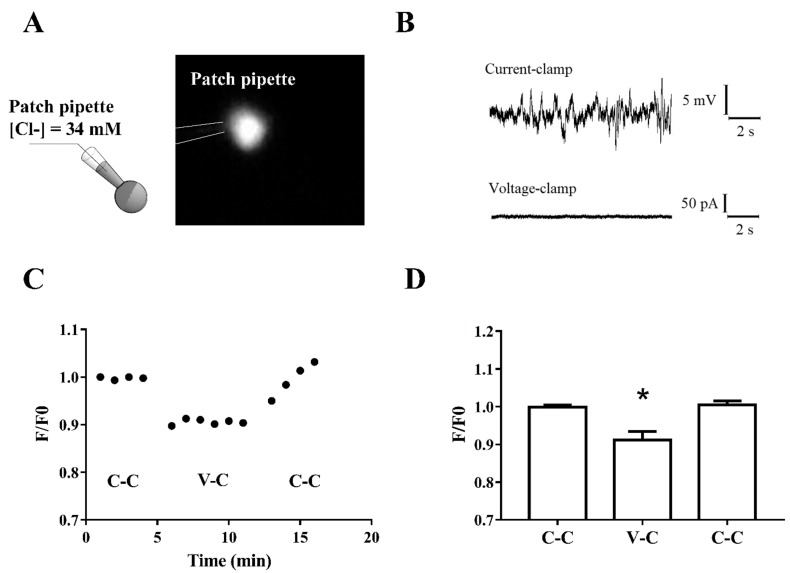
Membrane potential oscillations regulate the intracellular calcium levels. (**A**) Representation of single-cell Ca^2+^ imaging method and fluorescence image of a recorded immature neuron. The intracellular solution contained 34 mM Cl^-^ and 100 µM Fluo4. (**B**) Resting membrane potential oscillations recorded in current-clamp mode (top trace) and low-noise voltage-clamp recording at the same potential in the same cell (bottom trace). (**C**) Representative course of cell body fluorescence in a recorded immature neuron obtained by switching recording mode from current-clamp (C-C) to voltage-clamp (V-C) and vice versa. (**D**) Significative decrease of [Ca^2+^]_I_ obtained by switching from current-clamp mode to voltage-clamp mode; the reduction reversed by switching back to current-clamp mode (RM one-way ANOVA F (2, 5) = 15.28 *p* < 0.01, Tukey’s posthoc test (*) V-C vs. both C-C, *p* < 0.05).

Cell body fluorescence changes (Figure 3A) were analyzed by switching the recording mode from current-clamp to voltage-clamp, setting the membrane potential at the value measured immediately after the establishment of whole-cell configuration. Low noise recordings were obtained in voltage-clamp mode, due to the poor or absent spontaneous synaptic activity. On the contrary, current-clamp mode was characterized by high noise recordings with robust hyperpolarizing and depolarizing membrane potential oscillations (Figure 3B). Interestingly, cell body fluorescence decreased significantly switching from current-clamp mode to voltage-clamp mode, and it fully recovered, switching back to current-clamp mode (Figure 3C,D).

### 2.3. Membrane Channels Involved in Resting Membrane Potential Oscillations

Resting membrane potential oscillations were investigated by perfusion bath application of specific channel/receptor agonists and blockers.

**Figure 4:**
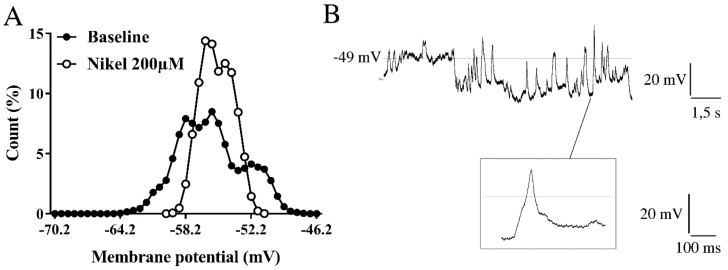
Membrane potential oscillations in immature granule cells. (**A**) Representative graph of membrane potential noise distribution recorded in immature neurons before and after 200 µM nickel application to the bath perfusion. (**B**) Robust spontaneous hyperpolarizing events recorded in current-clamp mode in immature neuron, showing a mean resting membrane potential of −49 mV. A T-type VDCC spike-like event is enlarged in the box.

To evaluate if membrane potential oscillations could be linked to intracellular Ca^2+^ level changes, 200 µM Ni^2+^, a T-type VDCC blocker, was applied during immature granule cell recordings. A significant decrease in both membrane noise and intracellular Ca^2+^ concentration was found, without any alteration of the resting membrane potential mean value (Figure 4A, Table 1).

Considering that NMDARs and calcium-permeable AMPARs could represent an additional source of calcium, NMDAR and AMPAR blockers were applied to disclose a possible involvement of glutamate ambient in membrane noise induction. The addition of both 50 µM AP5 (an NMDAR blocker) and 10 µM CNQX (an AMPAR blocker) to the recording solution did not affect resting membrane potential noise and intracellular calcium level as well (Table 1).

During recordings, immature neurons showed robust hyperpolarizing bursts that, due to their amplitude, were assumed to be mediated by the BK channel opening (Figure 4B). Thus, a specific blocker of the BK channel, Paxilline (10 µM), was added to the perfusion bath. Both membrane potential oscillations and intracellular calcium levels significantly decreased (Table 1).

Then, to test the possible implication of GABA_A_R activation in intracellular calcium level variations and in membrane noise, GABA (10 µM) or muscimol (10 µM) were applied to the bath solution during immature cell recordings. In both experimental conditions, a significant reduction in intracellular calcium concentration and membrane potential noise was found (Table 1). During agonist application, RMP was close to the chloride equilibrium potential, thus inducing a slight membrane depolarization, which was not able to increase intracellular calcium level, probably due to T-Type VDCC inactivation. However, GABA_A_R opening decreased IR (data not shown), thus shunting membrane currents and dampening resting membrane potential oscillations.

### 2.4. Hippocampal Circuit Activity and Intracellular Calcium Level in Immature Neurons

The effect of the physiologically released GABA neurotransmitter on very immature neurons was evaluated by stimulating MPP. To obtain a constant extracellular GABA concentration, low-frequency stimulation was applied to MPP (3 min, 2.5 Hz). GABAergic responses could be evoked by immature synapse activation onto newborn neurons or by GABA spillover from mature synapses close to the recorded neuron.

**Figure 5:**
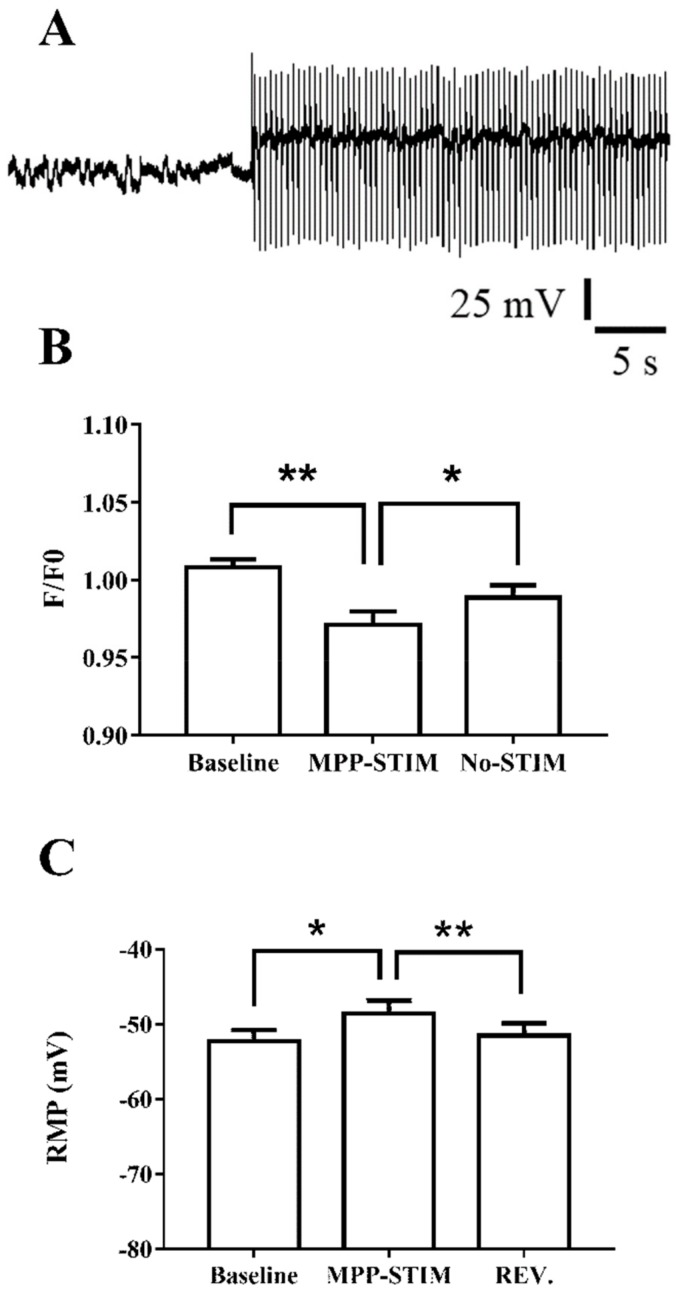
GABA_A_R activation by MPP stimulation regulates intracellular calcium concentration. (**A**) Typical response evoked by MPP stimulation (2.5 Hz) recorded in current-clamp mode. MPP stimulation induced a constant slight depolarization shifting membrane potential close to the Cl^-^ equilibrium. Even if the trace is disturbed by artifacts, it is possible to note the reduction of potential oscillations during stimulation. (**B**) Prolonged MPP stimulation (3 min) induced a significant and reversible decrease of [Ca^2+^]_I_ and membrane depolarization, as well (**C**). F/F0, RM one-way ANOVA F (2, 12) = 11.38 *p* < 0.01, Tukey’s post hoc test (**) baseline vs. MPP-STIM *p* < 0.01, (*) MPP-STIM vs. No-STIM *p* < 0.05. RMP, RM one-way ANOVA F (2, 12) = 9.374 *p* < 0.01, Tukey’s post hoc test (*) baseline vs. MPP-STIM *p* < 0.05, (**) MPP-STIM vs. No-STIM *p* < 0.01.

MPP stimulation induced a slight depolarization (thus moving resting membrane potential towards the chloride equilibrium potential), and decreased intracellular calcium level as well (Figure 5A–C). This result was consistent with those obtained by applying GABA or muscimol to the perfusion bath. The noise analysis was not performed due to the recording contamination by stimulus artifact, but a reduction of membrane potential oscillations during stimulation could be appreciated (Figure 5A).

### 2.5. Interaction between BK Channels and T-Type Voltage-Dependent Ca^2+^ Channels

**Figure 6:**
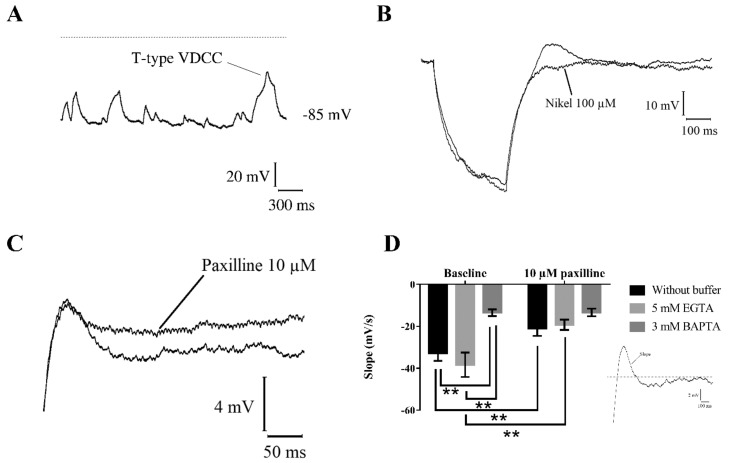
BK channels and T-type VDCC interaction. (**A**) Hyperpolarizing spontaneous burst recorded in current-clamp mode in immature neuron with resting membrane potential mean value of −52 mV. (**B**) Ni^2+^-sensitive T-type VDCC spikes induced by hyperpolarizing current steps starting from resting membrane potential. (**C**) Paxilline-sensitive after-hyperpolarization induced by T-type VDCC spike. (**D**) BK-mediated after-hyperpolarization, quantified as the slope of calcium spike repolarization (inset in the graph), recorded using different intracellular calcium chelators. Two-way RM ANOVA with *Paxilline treatment* and *Intracellular buffer* as independent variables; *Paxilline treatment* F (1, 18) = 26.82 *p* < 0.01, *Intracellular buffer* F (2, 18) = 9.256 *p* < 0.01. Tukey’s multiple comparisons test (**) baseline vs. 10 µM paxilline without buffer *p* < 0.01, (**) baseline vs. 10 µM paxilline 5 mM EGTA *p* < 0.01, (**) baseline without buffer vs. baseline 3 mM BAPTA *p* < 0.01, (**) baseline 5 mM EGTA vs. baseline 3 mM BAPTA *p* < 0.01.

As mentioned above, all recorded cells showed a robust membrane noise in current-clamp mode, and, in some of them, it was also possible to distinguish a hyperpolarizing burst showing the typical shape of the RC circuit response to square hyperpolarizing current (Figure 6A). Furthermore, at the end of the hyperpolarizations, a depolarizing event similar to the T-type calcium spike (Figure 6A) was frequently detectable. In principle, this is consistent with the fact that in immature neurons, showing depolarized resting membrane potential, the only way to open T-Type calcium channels relies on hyperpolarizing events able to remove ion channel inactivation. Therefore, to verify this possibility, spontaneous hyperpolarization was simulated using increasing hyperpolarizing current steps (300–600 ms) in current-clamp mode, and a Ni^2+^-sensitive calcium spike was elicited in all recorded immature neurons (Figure 6B).

Interestingly, calcium spike was followed by a hyperpolarization (after-hyperpolarization), which suggested a possible involvement of calcium-activated potassium channels. To test this hypothesis, 10 µM paxilline, a specific BK channel blocker, was applied to the recording bath. Paxilline fully abolished the after-hyperpolarization, thus confirming the involvement of this type of potassium channels (Figure 6C).

BK channel requires high intracellular Ca^2+^ concentration for opening, and this may occur when the channel is closely apposed to the calcium channels forming a heteromeric complex [16]. To verify this possibility, different intracellular calcium chelators were used during current-clamp recordings. T-Type-mediated calcium spikes were able to elicit paxilline-sensitive hyperpolarization in absence of intracellular calcium buffers or in presence of 5 mM EGTA (Figure 6D). In sharp contrast, fast calcium buffer BAPTA (3 mM) added to the intracellular solution, completely abolished calcium-induced hyperpolarization sensitive to paxilline (Figure 6D), thus suggesting a physical and functional coupling between BK channels and T-Type VDCCs.

**Figure 7:**
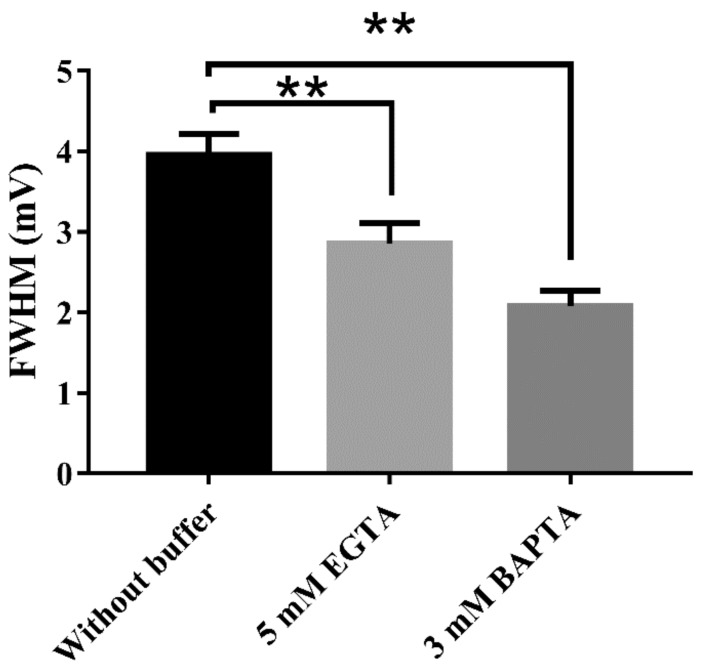
Resting membrane potential oscillations and intracellular calcium concentration. Membrane potential oscillations decreased by increasing calcium buffer capacity. One-way ANOVA F (2, 17) = 16.28 *p* < 0.01, Tukey’s multiple comparisons test (**) without buffer vs. 5 mM EGTA *p* < 0.01, (**) without buffer vs. 3 mM BAPTA *p* < 0.01.

Finally, the analysis of membrane noise revealed that membrane potential oscillations were attenuated by increasing intracellular calcium buffer capacity (Figure 7).

## 3. Discussion

**Figure 8:**
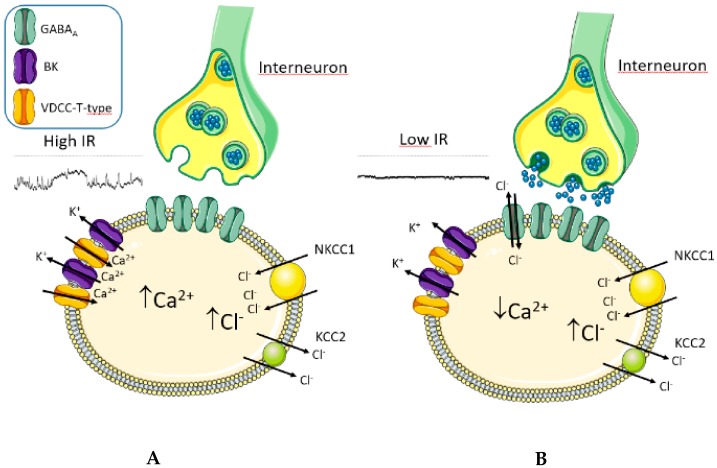
Schematic illustration showing the implication of BK channels, T-type VDCC, and GABA_A_R in intracellular calcium level regulation. (**A**) When the extracellular GABA concentration is low, GABA_A_Rs are closed, immature neurons have a very high IR, and generate membrane potential oscillations through BK and T-type calcium channel activity. Specifically, robust BK-induced hyperpolarizations elicit T-type VDCC voltage sensor activation and gate opening, which increases calcium entry. In turn, calcium influx quickly activates BK channels, due to their physical interaction (according to the literature [17]), inducing hyperpolarizations. This mechanism is facilitated by low calcium buffer levels present in immature neurons. (**B**) When the extracellular GABA concentration increases, GABA_A_Rs are activated, inducing membrane current shunt. BK-induced hyperpolarization is damped, preventing T-type VDCC opening. Therefore, an increased hippocampal activity through GABA_A_R action can promote a lowering of intracellular calcium level. This figure was prepared using the Neuroscience-PPT-Toolkit-Suite of Motifolio Inc., USA, and Servier Medical Art (https://smart.servier.com/).

In the present work, spontaneous membrane potential oscillations observed in very immature neurons of adult rat dentate gyrus were related to intracellular calcium level alterations and their modulation by GABA activity was investigated. The main results we found were the following: (i) resting membrane potential oscillations were able to elicite calcium entry into the cell, thus increasing intracellular calcium concentration; (ii) BK channels and T-Type VDCCs were involved in generating membrane potential oscillations; (iii) GABA_A_R activation was able to decrease intracellular calcium level, probably shunting membrane currents and dampening resting membrane potential oscillations. Altogether, these findings outline a novel mechanism through which GABA regulates intracellular calcium homeostasis in rat adult-born granule neurons at a very early stage of their maturation (Figure 8).

Our findings seem not to be consistent with the well-documented hypothesis that GABAergic activity induces intracellular calcium elevation in immature neurons [5,18,19]. Such discrepancy may conceivably be explained, taking into account that in our experimental model, the recorded developing granule neurons show a resting membrane potential, definitely more depolarized compared to T-type VDCC threshold activation, thus inducing calcium channel inactivation. Therefore, although GABA is depolarizing, moving resting membrane potential towards the chloride equilibrium potential, there are not the electrical conditions needed for T-type VDCC opening and the resulting calcium influx. Of course, we cannot rule out the possibility that GABAergic depolarizing activity may activate T-type VDCCs when it occurs in conjunction with a hyperpolarizing spontaneous event or in less immature neurons showing a resting membrane potential more hyperpolarized compared to T-type VDCC threshold, as we previously demonstrated (Class 2 subclass III described in [2]).

Concerns on technical caveats in patch-clamp recordings of resting membrane potentials may arise considering, in particular, the high IR of immature neurons that might introduce an error leading to underestimating RMP values. However, this error can be avoided by performing a high resistance seal onto the cell membrane (approximately 10-times higher than cell IR), as described in the literature [20,21,22]. Therefore, we believe our results are reliable because of the high resistance seals we obtained onto the membrane of the recorded immature neurons, as recommended in the literature. In addition, the maturation stage of newborn neurons we analyzed in this work was earlier than that of immature neurons considered in the investigations performed by other authors [10,20,21,22], as can be inferred comparing their functional features.

The passive membrane properties and the depolarized resting membrane potential of the examined immature neurons induce the activation of a mechanism that generates membrane potential oscillations and calcium influx by involving BK channels. The BK channel is characterized by an exceptionally large single-channel conductance, and it can be synergistically activated by membrane depolarization and elevation of intracellular Ca^2+^ concentration, resulting in membrane repolarization and voltage-dependent Ca^2+^ channel closing to reduce Ca^2+^ entering the cell. However, the BK opening probability may be increased by the presence in the channel structure of β4 subunits, being able to shift the voltage dependence to more negative potentials [23,24], and by the proximity of a calcium source. The expression of BK-β4 subunits in the plasma membrane of dentate granule cells was demonstrated by Brenner and colleagues [25]. This result, together with our present findings, indicating a clustering between the BK channel and T-type VDCC, allow us to assert that the conditions to increase the BK channel opening probability exist in the immature neurons. In addition, the low intracellular calcium buffer, described in newborn dentate granule cells by Stocca and colleagues [26], represents a further favorable condition for BK opening.

It is known that BK channels in mature dentate granule cells form complexes with NMDA receptors [27], and this structural and functional coupling might also occur in immature neurons. In line with this, we found a robust BK current after local NMDA or glutamate application onto the recorded immature neurons, but ambient glutamate levels were not able to open NMDARs in our experimental conditions. Nevertheless, we cannot rule out a role of these receptors on membrane potential oscillations and intracellular calcium regulation in the intact brain.

Concerning the meaning of the electrophysiological and molecular mechanism we highlighted, it has to be taken into account that the features of the immature neurons examined in this work indicate their low probability of sending information in the hippocampal circuit, especially because of a very high threshold and low amplitude of action potential (see Figure 1 and [2]). Therefore, the new granule neurons at this early stage of maturation, even though contacted, may not play a significant role in the hippocampal function. Thus, phasic and tonic GABA activity related to hippocampal activation might affect immature neuron developmental process, such as dendritic arborization, neuronal migration [28,29,30], as well as cell survival, likely by influencing intracellular calcium fluctuations.

Along this line of reasoning, we previously demonstrated in the adult rat dentate gyrus that hippocampal circuitry activation, related to physical activity and behavioral experiences, promotes anticipation of GABA synaptogenesis and T-type VDCC appearance onto 1-week-old newborn neurons [9,14] and that the survival of newborn cells was enhanced [31]. Therefore, considering the present findings, we may speculate that GABA input activation, related to hippocampal function, prevents newborn cell death by modulating intracellular Ca^2+^ transients. Indeed, based on the mechanism here described, GABA_A_R activation stabilizes the membrane potential at the Cl^-^ equilibrium potential, that in immature neurons is depolarized compared to the RMP, thus quenching the membrane potential oscillations generated by the coordinated activity of BK channel and T-type VDCC complexes during which calcium influx occurs.

In conclusion, our findings provide a significant contribution to understanding how adult-generated new neurons in the early stage of their maturation can regulate intracellular calcium levels, and highlight an unconventional role of GABA activity in intracellular calcium homeostasis. The electrophysiological and molecular mechanism here defined could also occur in immature neurons generated during brain development, which show functional features very similar to those of maturating neurons we have considered in this work and that have been described by Pedroni and colleagues in developing dentate gyrus [32].

Finally, future experiments will be required to verify the implication of the described mechanism in promoting newborn neurons survival.

## 4. Materials and Methods

### 4.1. Animals and Slices Preparation

We used Sprague–Dawley male rats (6–8-week-old, *n* = 25) in accordance with the Italian law on animal experimentation (D.lgs. 26/2014; research project permitted with authorization N. 465/2015-PR by Italian Ministry of Health). Animals were housed two per cage, where access to food and water was free. Room temperature was kept at 21 ± 1 °C, humidity was 50% + 5%, and light/dark cycle was 12–12 h (light on at 7.00 a.m.). Rats did not undergone any procedure, but were anesthetized with ketamine (65 mg/kg b.w.) and killed by decapitation. Brain was rapidly removed, and 400 µm-thick hippocampal transversal slices were prepared, as previously described [33]. Slices were recovered in oxygenated artificial cerebrospinal fluid (ACSF, containing in mM: 125 NaCl, 2.5 KCl, 1.3 NaH_2_PO_4_, 25 NaHCO_3_, 2 CaCl_2_, 1.3 MgCl_2_, 1.3 Na^+^ ascorbate, 0.6 Na^+^ pyruvate, 10 dextrose; pH = 7.4; 320 mOsm) for at least 1 h at room temperature, and then were transferred to the recording chamber where slices were continuously perfused (2 mL/min) with the saline solution. Patch-clamp recordings, in whole-cell or perforated configuration, were carried out under visual guidance using a Zeiss Axioskop microscope (Carl Zeiss International, Milan, Italy) equipped with an infrared light-sensitive camera connected to a monitor.

### 4.2. Patch-Clamp Recordings

Whole-cell recordings were performed by using patch pipettes filled with an internal solution that mimicked the intracellular environment of immature neurons (high intracellular chloride concentration assessed by perforated patch recordings). This solution contained in mM: 100 potassium gluconate, 26 KCl, 8 NaCl, 0.2 EGTA, 10 HEPES, 3 Mg_2_ATP, 0.3 GTP (pH = 7.2; 290 mOsm). In some experiments, intracellular calcium buffer concentration was increased by using 5 mM EGTA or 3 mM BAPTA. To minimize errors on membrane potential measure, we performed high resistance seals on the cell membrane (above 100 GΩ). Immature granule cells belonging to Class 2 subclass I, according to our previously reported classification [2], were electrophysiologically identified to be included in the present work. Cell access was obtained in the voltage-clamp mode, and resting membrane potential (RMP) was evaluated immediately upon break-in. Membrane potentials were measured with correction for liquid junction potentials. Passive membrane properties, such as input resistance (IR) and capacitance (C), were calculated in response to a 300 ms, 5 mV hyperpolarizing pulse. To evaluate cell excitability, we measured membrane potential in response to depolarizing and hyperpolarizing current pulses (5 pA steps). T-Type VDCC opening was obtained in current-clamp mode, using two different ways: (i) by depolarizing current pulses (300 ms) starting from a holding potential of about −80 mV; and (ii) by hyperpolarizing pulses (300–600 ms) starting from resting membrane potential (about −50 mV). Synaptically evoked responses were elicited using a bipolar stimulating electrode pulled from theta capillary (World Precision Instruments) and filled with ACSF solution. The bipolar electrode was placed in the molecular layer to activate the medial perforant pathway. Postsynaptic currents (PSCs) were evoked with an interstimulus frequency of 0.033 Hz for synaptic characterization or with 2.5 Hz (3 min) stimulation to obtain prolonged and constant GABA release from hilar interneurons.

Patch-clamp recordings in a perforated configuration were carried out as previously described [10] by using an internal solution containing in mM: 134 KCl, 0.2 EGTA, 10 HEPES, and 5 µg/mL gramicidin.

### 4.3. Calcium Imaging

Calcium imaging recordings were performed in whole-cell configuration using Fluo-4 pentapotassium salt, a calcium indicator dye (Thermo Fisher, Waltham, MA, USA), as previously described [34]. Briefly, the experiments were carried out using a Zeiss Axioskop microscope (Carl Zeiss International, Italy) equipped with a 40× water immersion objective and the Orca Flash 4.0 CCD camera (C11440, Hamamatsu, Japan), an Axopatch-200B amplifier (Axon Instruments, San Jose, CA, USA) and WinFluor software (Strathclyde Imaging Software V 3.8.7, John Dempster, University of Strathclyde, UK). Patch pipettes were filled with an intracellular solution containing in millimolar: 100 potassium gluconate, 26 KCl, 8 NaCl, 0.2 EGTA, 10 HEPES, 3 Mg_2_ATP, 0.3 GTP, 100 µM Fluo4 (pH = 7.2; 290 mOsm). Fluorescence images (100 × 100 pixels) were acquired at 100 Hz frequency using a FITC excitation filter of 450–490 nm and fluorescence values were expressed as F/F0 where F0 is the fluorescence at the start of recording. Fluorescence evaluation started immediately after the break-in and during the loading phase, F0 has been monitored. The steady-state was reached after about 15 min. Moreover, once the cells were fully loaded with Fluo-4, fluorescence changes in response to different channel/receptor agonists and blockers and MPP stimulation were evaluated.

During calcium imaging experiments, we recorded membrane potential in current-clamp mode, keeping the cell at its resting membrane potential. Recorded values were displayed as an amplitude histogram, and the full width at half maximum (FWHM) was adopted as a measure of membrane potential oscillation.

### 4.4. Drugs Application

Drugs were applied to the perfusion bath or through puff apparatus, as previously described [15]. Verapamil (20 µM), nickel (100 µM or 200 µM), TEA (2 mM), muscimol (10 µM), bicuculline methiodide (BMI, 10 µM), glutamate (100 µM), NMDA (300 µM), AP5 (50 µM), CNQX (10 µM) were purchased from Sigma, Italy. Paxilline (10 µM) was purchased from Tocris Bioscience UK, and TTX (500 nM) from Alomone Labs, Israel.

### 4.5. Statistical Analysis

Data were expressed as mean ± SEM. Differences between the experimental treatments were statistically evaluated by appropriately applying RM one-way ANOVA or two-way RM ANOVA followed by Tukey’s posthoc test. The significance threshold was established at *p* < 0.05.

## Figures and Tables

**Table 1:** Effects of channel/receptor agonist and blocker application on FWHM, F/F0, and RMP in the recorded immature neurons.

FWHM (mv)															
Treatment	Descriptive Statistics							RM-One-Way Anova		Tukey HSD					
	*n*	BL		TREAT		REV		*F(df)*	*p*	BL *vs.* TREAT		BL *vs.* REV		TREAT *vs.* REV	
		Mean	SEM	Mean	SEM	Mean	SEM			Mean Diff.	*p*	Mean Diff.	*p*	Mean Diff.	*p*
Nikel 200 µM	7	3.8	0.5	2.4	0.4	3.6	0.5	F(2,6) = 10.98	0.005	1.71	0.03	0.20	*ns*	−1.51	0.02
AP5 50 µM	6	4.9	0.7	4.8	0.7	4.2	0.6	F(2,5) = 0.79	*ns*	0.11	*ns*	0.66	*ns*	0.55	*ns*
CNQX 10 µM	5	4.8	0.9	4.7	0.9	4.2	0.8	F(2,4) = 0.56	*ns*	0.08	*ns*	0.64	*ns*	0.56	*ns*
Paxilline 10 µM	8	2.5	0.1	1.8	0.1	2.7	0.2	F(2,7) = 12.71	0.004	0.77	0.001	−0.12	*ns*	−0.9	0.01
GABA 10 µM	10	5.2	0.5	3.5	0.3	4.6	0.5	F(2,9) = 4.01	0.049	1.68	0.02	0.54	*ns*	−1.14	*ns*
Muscimol 10 µM	8	5.6	0.6	3.2	0.3	5.6	1.0	F(2,7) = 4.97	0.04	2.36	0.007	−0.03	*ns*	−2.4	*ns*
F/F0															
Nikel 200 µM	10	1.01	0.01	0.91	0.02	0.94	0.03	F(2,9) = 9.63	0.002	0.09	0.004	0.06	*ns*	−0.03	*ns*
AP5 50 µM	7	1.01	0.01	1.01	0.02	1.03	0.03	F(2,6) = 0.17	*ns*	0.00	*ns*	−0.01	*ns*	−0.01	*ns*
CNQX 10 µM	5	1.00	0.01	1.02	0.02	1.07	0.05	F(2,4) = 0.83	*ns*	−0.02	*ns*	−0.06	*ns*	−0.04	*ns*
Paxilline 10 µM	8	1.02	0.01	0.77	0.05	0.7	0.07	F(2,7) = 17.02	0.001	0.24	0.008	0.31	.007	0.07	*ns*
GABA 10 µM	6	0.99	0.00	0.92	0.02	1.00	0.00	F(2,5) = 15.86	0.009	0.07	0.02	−0.01	*ns*	−0.07	.02
Muscimol 10 µM	9	1.00	0.01	0.88	0.03	0.93	0.06	F(2,8) = 4.42	0.04	0.14	0.01	0.06	*ns*	−0.07	*ns*
RMP (mv)															
Nikel 200 µM	8	−55.8	4.9	−55.4	5.3	−55.8	5.3	F(2,7) = 0.03	*ns*	−0.31	*ns*	0.06	*ns*	0.37	*ns*
AP5 50 µM	6	−52.3	2.6	−52.0	2.5	−55.2	3.2	F(2,5) = 2.31	*ns*	−0.33	*ns*	2.83	*ns*	3.16	*ns*
CNQX 10 µM	5	−52.8	2.0	−52.6	2.2	−51.8	2.1	F(2,4) = 2.31	*ns*	−0.20	*ns*	−1.00	*ns*	−0.8	*ns*
Paxilline 10 µM	6	−49.7	2.1	−41.8	4.8	−42.8	5.7	F(2,5) = 4.20	*ns*	−7.83	*ns*	−6.83	*ns*	1.00	*ns*
GABA 10 µM	8	−49.3	2.2	−41.1	1.4	−53.3	1.8	F(2,7) = 19.56	0.0004	−8.12	0.02	4.00	*ns*	12.13	0.0001
Muscimol 10 µM	8	−50.1	2.5	−41.0	1.3	−52.5	1.7	F(2,7) = 14.77	0.001	−9.12	0.015	2.37	*ns*	11.5	0.0003
